# Current Status on Population Genome Catalogues in different Countries

**DOI:** 10.6026/97320630016297

**Published:** 2020-04-30

**Authors:** Rahul Kumar, Sandeep Kumar Dhanda

**Affiliations:** 1 Institute for Cancer Genetics, Columbia University Irving Medical Center, New York, NY10032, USA; 2 Department of Oncology, Saint Jude Children's Research Hospital, Memphis, TN 38105, USA

**Keywords:** Genomes catalogues, clinical genomics, data initiatives, Bioinformatics

## Abstract

Genomics has become indispensable for research in the last two decades. Completed and ongoing genome projects such as the UK Biobank, ICGC, TCGA and GenomeAsia100K have helped to understand several life-threatening diseases like cancer. Such initiatives from different countries have offered genomics-based diagnostics along with glues for therapies towards personalized healthcare. The Indian Agencies has started initiatives to catalogue the genome sequences of 20,000 individuals. The Department of Biotechnology (DBT) along with other scientific agencies has plans to sequence 10,000 healthy individuals and 10,000 diseased individuals. The Council of Scientific and Industrial Research (CSIR) also developed the "IndiGen" genome project where genome sequences for 1008 individuals are made available in Phase I. This will enable the development of a genome catalogue to introduce novel genomics-based clinical applications in future healthcare plan.

## Global genomics initiatives:

**Figure 1:**
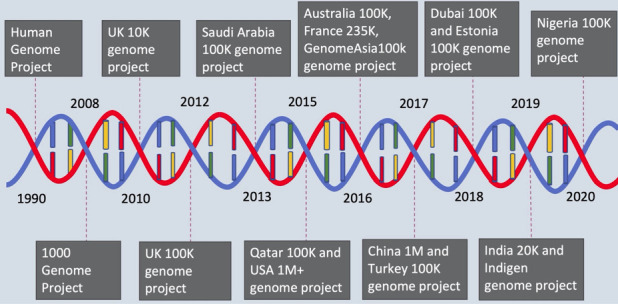
Timeline for human population genomic catalogue initiatives to the years the projects were launched. The authors declare that this image is their original work.

Since the completion of the first human genome project, the cost of sequencing went down dramatically. This made it possible to sequence the genome of a large number of individuals to study population and disease genetics. Countries like Australia, China, England, Estonia, France, Japan, Qatar, Saudi Arabia and Turkey have launched their respective genomics projects to map the genomic landscape in their respective population during the last decade (Figure 1). The DBT, India also launched a human genome catalogue project to sequence 20,000 individuals. This project consists of two phases. The first phase will sequence 10,000 normal individuals to map the genetic variability existing in the Indian population. The second phase will sequence 10,000 diseased individuals. The diseases will include cancer, heart disease, diabetes and several others. Genomics data from these two phases will eventually be compared to identify the putative genetic causes of diseases. The participation of private sectors in genome sequencing is highly appreciated. The project collaboration between MedGenome Labs, Private Ltd, India and NANYANG Technological University (NTU), Singapore collected genome sequences for 598 Indians [1].

## Goals for Genome Catalogue:

Genomics projects come with great promises. The participation from India in genome projects is highly appreciated. Data will help in the mapping of genetic variability among the Indian population with the global population. Genetic variation will be used in predictive medicine. Novel variants can be linked to the predisposition of a particular disease in a specific population e.g. association of V122I variant with heart failure in African or Hispanic/Latino individuals using machine learning techniques aided deep learning [2]. Efficiency of a drug is not equivalent in different individuals and identification of novel variants that may affect the drug metabolism and pharmacokinetics will enable personalized medicine [3]. Novel variants associated with diseases like cancer risk will help in genetic counselling of Individuals for taking suitable actions. Drug resistance is an impediment in disease combat and care. This will be explained by the heterogeneity. These big-scale genomics projects will enable the understanding of heterogeneity [4].

## Indian Genomics Initiatives:

India announced the first Indian genome data in 2009. The Indian government announced a much-coveted genomics project called 'IndiGen: Genomics for public health in India' in 2019 and made available at https://indigen.igib.in/. The genome sequence of 1008 individuals have been completed as part of phase I. These 1008 individuals represent the genetic variability in India. Individuals sequenced under this program will have full access to their genome information using an application. The future holds more promise in understanding the human genetics.

## Conclusion:

Efforts taken by governments towards enhancing the understanding of genomes are very promising. This will enable clinicians to tailor individualized treatments and offer genetic counselling on an individual basis. Despite these benefits, these efforts need to be scaled up in terms of the number of individuals sequenced to cover the vast diversity of the population. Data representation, catalogue models, storage platforms and security are a concern [5-6].The role played by government and non-government agencies in providing services with improved healthcare using known biomedical data is highly relevant.
